# A Case of Perforated Duodenal Diverticular Stone

**DOI:** 10.7759/cureus.38468

**Published:** 2023-05-03

**Authors:** Monis J Ahmed, Vijay C Vinod, Muhammad Umar Younis, Muhammad Shafique, Faisel Ikram

**Affiliations:** 1 General Surgery, Mediclinic City Hospital, Dubai, ARE; 2 Medicine, Queen's Hospital, London, GBR

**Keywords:** diverticular stone, duodenal fistula, rare entity, duodenal diverticula, duodenal perforation

## Abstract

Nontraumatic surgical emergencies constitute a major bulk of general surgical practice. Most of the cases seen fall under routine, but now and then, a surgeon or emergency physician is faced with an unusual diagnosis or a rarer presentation of a usual diagnosis. Sharing among peers their experiences with these outliers of practice helps spread knowledge and increases the experience pool. We share our experience of a 66-year-old female who presented to our emergency with upper abdominal pain of one-day duration.

## Introduction

Diverticula are a fairly common entity encountered in a general surgical practice with the majority of the encounters being limited to the colon. The duodenum is widely recognized as a distant second with incidence in the general population estimated to be around 22% [1]. As the majority are asymptomatic, even when encountered during routine endoscopies, they do not alter clinical management. However, once symptomatic, they can be a cause of both diagnostic and therapeutic dilemmas for the surgeons.

## Case presentation

**Figure 1:**
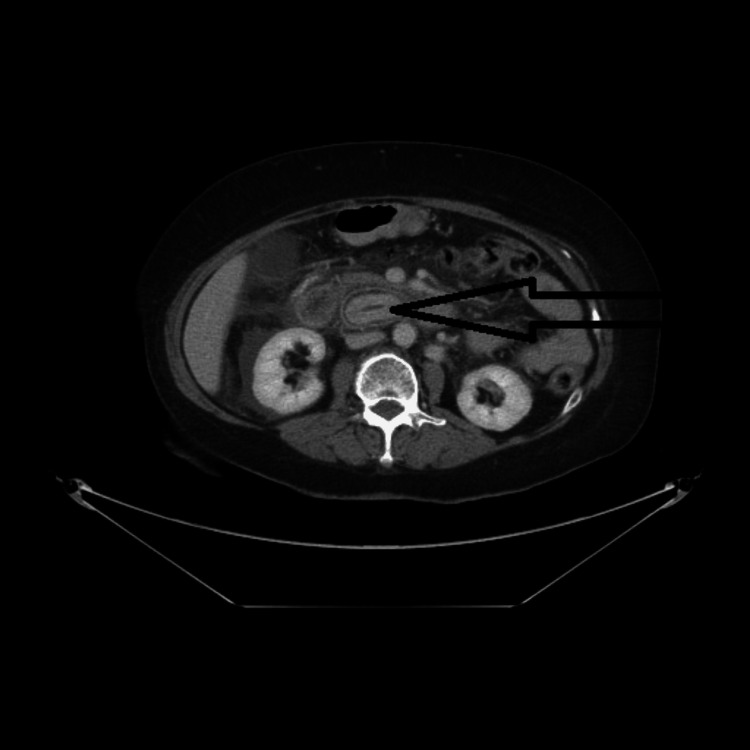
Axial image demonstrating retroperitoneal stone (arrow)

**Figure 2:**
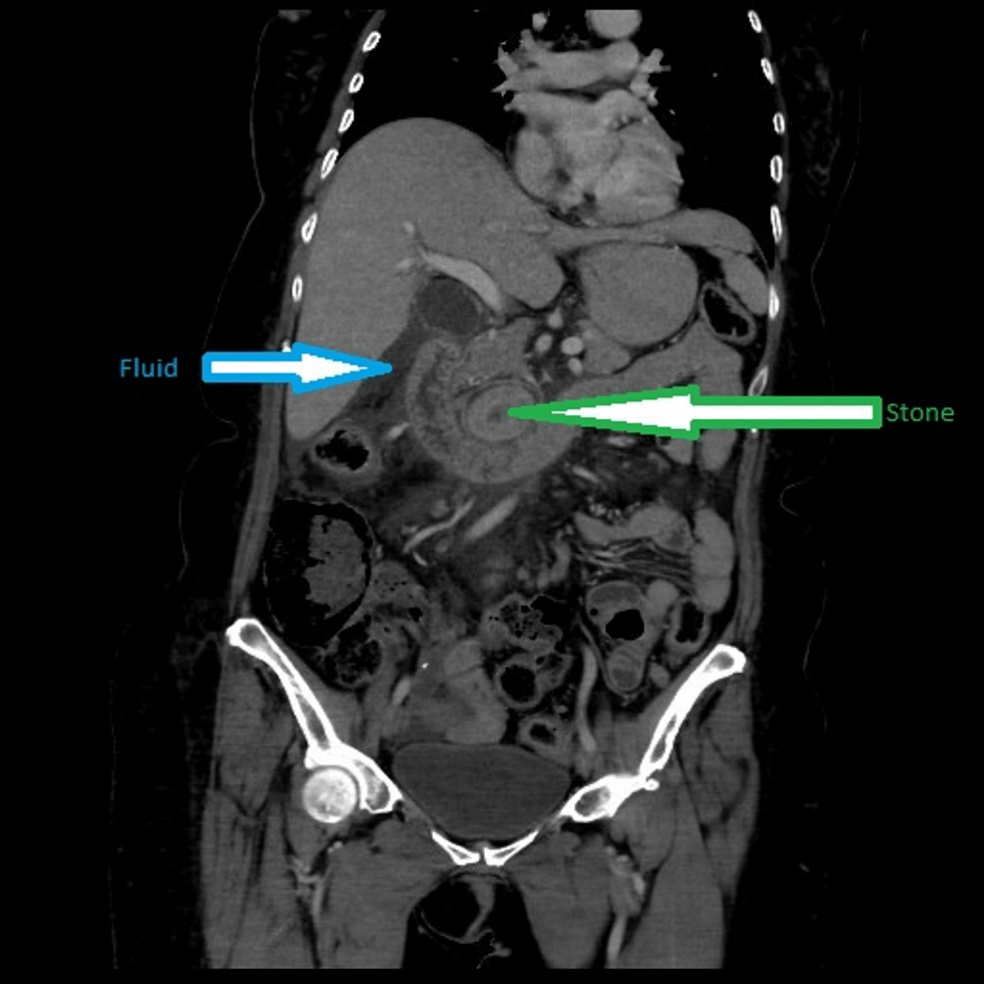
Coronal view demonstrating fluid (blue arrow) and stone (green arrow)

A 66-year-old female who has a history of acute-onset upper abdomen pain for one day was brought to the emergency department. Associated with nausea and vomiting, the pain was radiating to the back. The vomitus was not blood-stained, and the pain had no aggravating or relieving factors. There was no history of diarrhea, fever, chest pain, or shortness of breath, but she had a past history of gastric bypass done three years ago. The patient was known to have type II diabetes mellitus, hypertension, thalassemia minor, and dyslipidemia. There was no family history of inflammatory bowel disease or malignancy. On examination, the patient was in acute discomfort due to severe pain with a pain scale of 10/10. The patient was afebrile. Her blood pressure was 121/63 mmHg, and her heart rate was 63/minute, regular. Cardiovascular and respiratory system examination was normal. On abdominal examination, the patient had epigastric and periumbilical tenderness with guarding. Bowel sounds were heard normally. Initial investigations revealed random blood glucose of 7.8 mmol/L. White blood cells were 11.6 K/uL (normal range: 4-11 K/uL), neutrophils 9.52 K/uL (normal range: 1.8-7.7 K/uL), hemoglobin 11 gm/dL (normal range: 11.50-16 gm/dL), amylase 186 IU/L (normal range: 25-125 IU/L), lipase 325 U/L (normal range: 8-78 U/L), and C-reactive protein <0.40 mg/L (normal range: 0.40-5 mg/L). Liver function tests, troponin I, and renal function tests all were reported as normal. In view of elevated pancreatic enzymes and acute abdomen, computed tomography (CT) of the abdomen with intravenous and oral contrast was done. CT revealed multiple gallstones and a 4.4×2.4 cm radiopaque structure lying in the retroperitoneum (Figure 1). Extensive inflammatory changes with fluid were noted in the retroperitoneum and root of the mesentery. The common bile duct was normal with no calculus. There was no intrahepatic duct dilatation. There was some fluid seen around the liver and gallbladder (Figure 2). A provisional diagnosis of perforation of the biliary tract was made with no definitive site.

**Figure 3:**
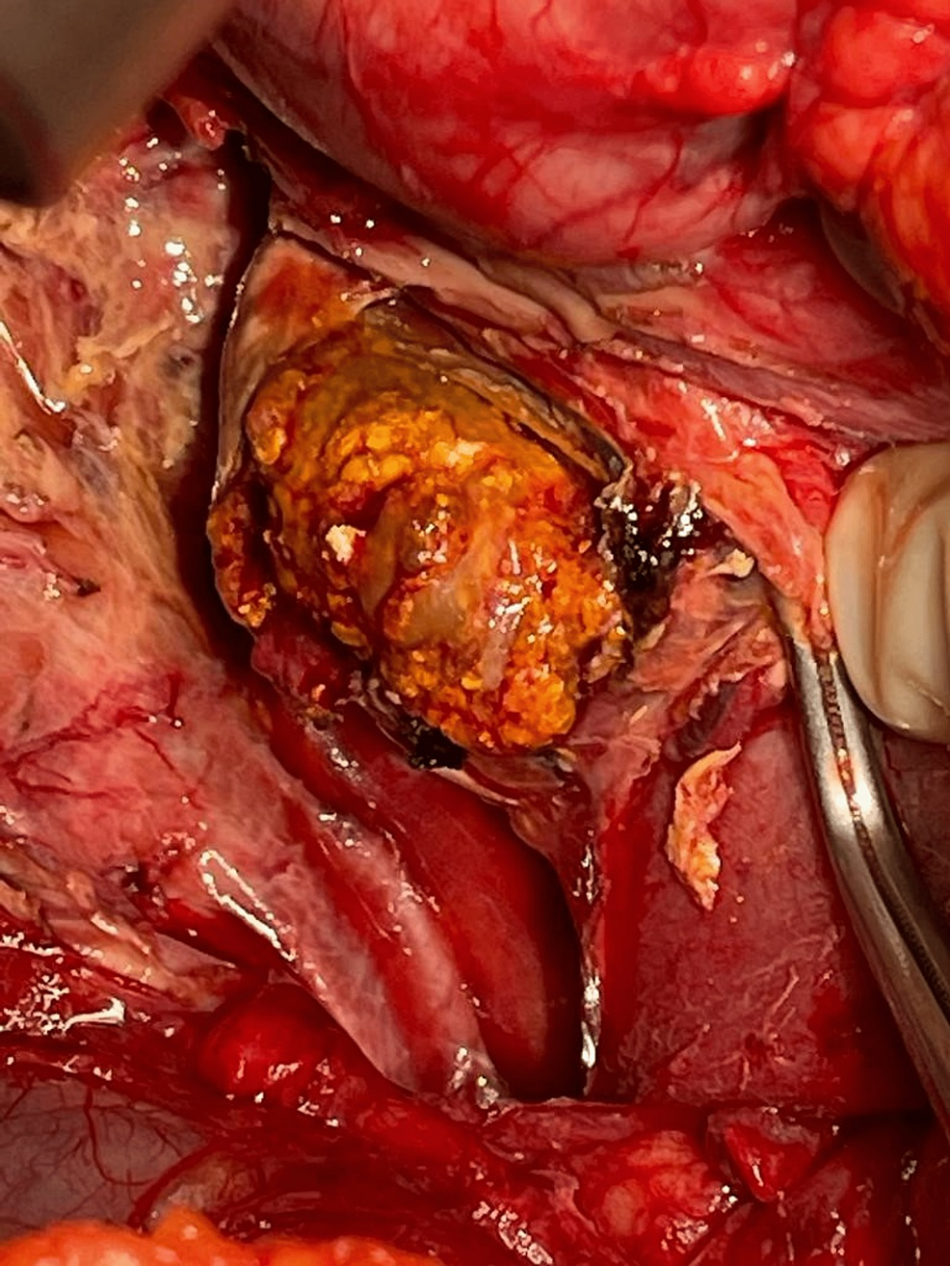
Intraoperative view

**Figure 4:**
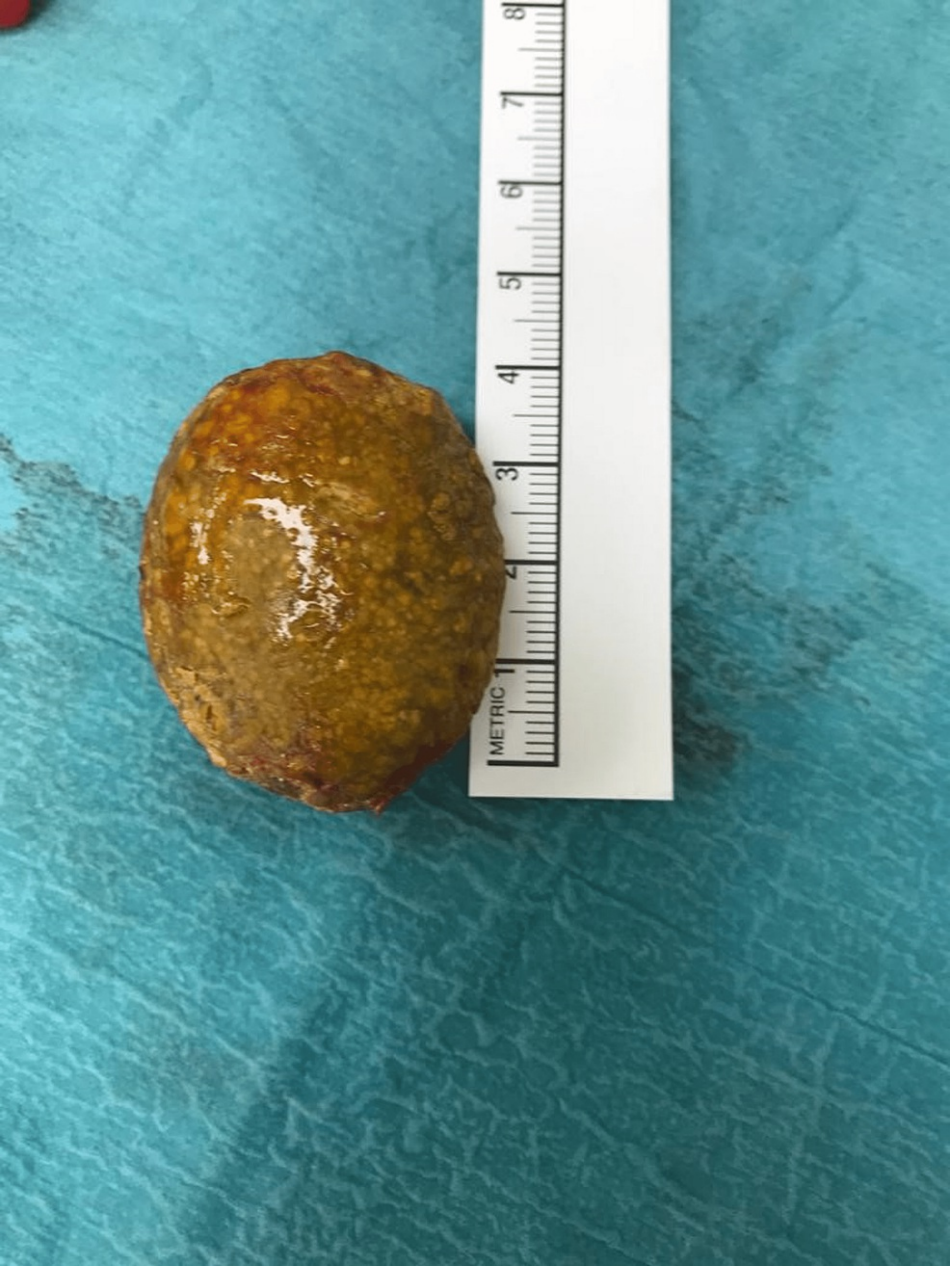
Stone size

The patient was taken to the operating theater. Initially, a laparoscopy was performed, and free, bile-stained fluid was identified around the liver and Morrison’s pouch. Extensive retroperitoneal edema and peritoneal inflammation were encountered, and a perforated duodenal diverticulum was identified arising from the second and third part, of which a large 4-cm stone was located in the retropancreatic region with extensive sloughing of surrounding tissues (Figure 3). Initial cholecystectomy was performed laparoscopically, but the latter part of the procedure requiring exposure to the retroperitoneum necessitated the conversion to laparotomy. The duodenum was primarily repaired with drain placement, and the stone was extracted (Figure 4). In view of multiple comorbidities, the patient was managed in the intensive care unit postoperatively. In addition to the routine analgesics and physiotherapy, she was managed by nasogastric drainage and total parenteral nutrition. She was initially discharged but later presented with a duodenal fistula. This was managed by the placement of radiologically guided drains. She required prolonged retention of the drains, but the output gradually subsided and she recovered well and was discharged.

## Discussion

Duodenal diverticula together with small bowel diverticula, as described by Eggert et al. [2] in 1982, are not usually symptomatic; however, they are frequently occurring anatomical entities. Diverticula are classified for description purposes as congenital and acquired, and the congenital are considered to be “true diverticula.” This is because they fulfill the definition of having all muscle layers of the wall included in the diverticular wall. The duodenal diverticula also fall in the same category if they are intraluminal. No definitive causal factor for duodenal diverticula has been implicated, and no relationship with a surgical history has been implicated to our knowledge.

As far as the clinical impact of these diverticula is concerned, Shuck and Stallion [3] stated that more than 90% of these diverticula are asymptomatic, and even those producing symptoms will hardly ever require surgical intervention. The same author in another series with Akhrass et al. [4] found that of the symptomatic small bowel diverticula, 79% were in the duodenum. In many instances, symptoms that are nonspecific are attributed to diverticula inaccurately.

Apart from nonspecific symptoms, Schnueriger et al. [5] implicated bleeding, inflammation, and perforation as potential complications in up to 5% of the cases. In the event of perforation, the mortality has been quoted to be up to 13% [6].

Stones in the duodenal diverticula are an even rarer occurrence. By itself, the perforation of duodenal diverticula has been reported less than 200 times in case reports according to Kimyaghalam et al. [7] in 2018. In a review of the literature performed by Thorson et al. [8] in 2012, they reported that the most common cause of perforation was diverticulitis (62%), and lithiasis only accounted for 10% of published cases reviewed. These figures highlight the rarity of the presentation of our patient.

The array of intervention choices, once a perforation is identified, may range from simple closure to the need of performing pancreaticoduodenectomy. Recently, there have been reports in the literature where a nonsurgical approach comprising fasting, nutritional support, and drainage has been successful [9]. However, one needs to be vigilant in taking such an approach as reported cases in the literature also demonstrate the futility or failure of such an approach [10]. Generally, as a logical surgical principle, the decision of conservative management will require taking many variables into consideration including, but not limited to, the availability of expertise, the clinical situation of the patient, and a definitive surgical plan in cases of non-progression or worsening of symptoms.

## Conclusions

The routine use of advanced radiology in emergency departments has decreased the need for surgical interventions with uncertain diagnoses. However, during the course of one’s career, there might be instances where despite scans showing a positive finding, a degree of diagnostic uncertainty is present, leading to unexpected surgical findings and diagnosis. This case is shared as a similar unique instance where the final diagnosis was not established until the operative findings were noted. Rare instances like this will help if kept in mind so that if the surgeon does encounter unexpected “surprises” during an operation, he can utilize various surgical options and can operate with a good surgical plan to deal with any complex scenario.
